# Gallic Acid-Laminarin Conjugate Is a Better Antioxidant than Sulfated or Carboxylated Laminarin

**DOI:** 10.3390/antiox9121192

**Published:** 2020-11-27

**Authors:** Marília Medeiros Fernandes-Negreiros, Lucas Alighieri Neves Costa Batista, Rony Lucas Silva Viana, Diego Araujo Sabry, Almino Afonso Oliveira Paiva, Weslley Souza Paiva, Raynara Iusk Araujo Machado, Francimar Lopes de Sousa Junior, Daniel de Lima Pontes, Jussier de Oliveira Vitoriano, Clodomiro Alves Junior, Guilherme Lanzi Sassaki, Hugo Alexandre Oliveira Rocha

**Affiliations:** 1 Department of Biochemistry, Universidade Federal do Rio Grande do Norte, Natal, Rio Grande do Norte 59.078-970, Brazil; marilia_negreiros16@yahoo.com.br (M.M.F.-N.); lucasalighieri@gmail.com (L.A.N.C.B.); rony_lucas@hotmail.com (R.L.S.V.); popoh.diego@gmail.com (D.A.S.); wdspaiva@gmail.com (W.S.P.); raynara_yusk@hotmail.com (R.I.A.M.); 2 Department of Biomedicine, Faculdade Nova Esperança (FACENE), Mossoró 59.628-000, Brazil; alminoafonso@yahoo.com; 3 Laboratório de Química de Coordenação e Polímeros-LQCPol, Instituto de Química, Universidade Federal do Rio Grande do Norte—UFRN, Natal-RN 59.078-970, Brazil; francimar_junnior@hotmail.com (F.L.d.S.J.); pontesdl@yahoo.com (D.d.L.P.); 4 Centro Integrado de Inovação Tecnológica do Semiárido (CiTED), Universidade Federal Rural do Semi-Árido, Mossoró 59.625-900, Brazil; ssier_6@hotmail.com (J.d.O.V.); clodomiro.jr@hotmail.com (C.A.J.); 5 Departament of Biochemistry, Federal University of Parana, Curitiba 81.531-980, Brazil; sassaki@ufpr.br

**Keywords:** β-glucan, brown seaweed, modified polysaccharide, dielectric barrier discharge, *Lobophora variegata*

## Abstract

A 12.4 kDa laminarin (LM) composed of β(1→3)-glucan with β(1→6)-branches was extracted from brown seaweed *Lobophora variegata* and modified via carboxylation using dielectric barrier discharge (LMC), conjugation with gallic acid (LMG), and sulfation (LMS). Analyses of the chemical composition of LMC, LMG, and LMS yielded 11.7% carboxyl groups, 1.5% gallic acid, and 1.4% sulfate content, respectively. Antioxidant activities of native and modified laminarins were assessed using six different in vitro methods. Sulfation stopped the antioxidant activities of LM. On the other hand, carboxylation improved cooper chelation (1.2 times). LMG was found to be a more efficient antioxidant agent than LM in terms of copper chelation (1.3 times), reducing power (1.3 times), and total antioxidant capacity (80 times). Gallic acid conjugation was further confirmed using Fourier transform infrared spectroscopy (FT-IR) and one- and two-dimensional NMR spectroscopy analyses. LMG also did not induce cell death or affect the cell cycle of Madin–Darby canine kidney (MDCK) cells. On the contrary, LMG protected MDCK cells from H_2_O_2_-induced oxidative damage. Taken together, these results show that LMG has the potent antioxidant capacity, and, therefore, potential applications in pharmacological and functional food products.

## 1. Introduction

Oxidative stress is a key cellular condition for the health of humans as well as other living species and is related to the number of reactive species in cells, especially reactive oxygen species (ROS). Proper ROS levels are essential for detoxification, cellular apoptosis, cell signaling processes, and generation of molecules such as prostaglandins and leukotrienes [1]. However, the presence of ROS in large quantities and/or in undesired cellular compartments may have detrimental effects on cells such as damages to the structural integrity of cells and molecules including DNA and proteins. ROS are also associated with inflammation [2], immunodeficiency, obesity, neurodegenerative diseases, multiple sclerosis, diabetes, cirrhosis, cancer [3], and urolithiasis [4]. Organisms produce endogenous antioxidant compounds that may be enzymatic or non-enzymatic antioxidants to combat the damage caused by ROS [5]. However, the amounts of these endogenous antioxidants are often insufficient to mount an effective response against reactive species. In humans, this is compensated by the absorption of exogenous antioxidants into the cells [6]. Antioxidant molecules are, therefore, often included in products of pharmaceutical, biomedical, and food industries. Nevertheless, since none of the commercial antioxidants show ideal antioxidant properties, there is always a need to find new antioxidants that can adapt to new situations and/or replace existing ones [7].

Seaweeds produce many different molecules with favorable properties, including antioxidants. The global demand for seaweed products that improve human health, including laminarin, continues to increase [8]. Laminarin is a polysaccharide that consists of β-D-glucan. Laminarin is synthesized in large quantities by different species of brown seaweeds [9], and previous studies have shown its potential as a promising drug carrier [10].

Polysaccharides were previously shown to be easily conjugated with other functioning molecules/clusters, and thereby obtain advantageous properties including antioxidant activities [11]. The use of environment-friendly techniques, termed “green methods”, is also favored for modification of polysaccharides [11]. Sulfation [12,13], conjugation with gallic acid [4,11], and dielectric barrier discharge (DBD) modification [14] are methods that increase the antioxidant activity of polysaccharides [15]. These methods are considered eco-friendly as well, as they involve the use of only a few or no environmentally harmful reagents.

The sulfation process yields polysaccharides containing sulfate groups. For sulfation to occur, it is necessary to attach sulfur trioxide to a polysaccharide hydroxyl group [16]. Several methods can be used for polysaccharide sulfation [17], yet most of them involve several steps and the use of many chemical reagents. One of the simplest and most efficient polysaccharide sulfation methods is to use sulfuric acid as a sulfate group donor and propanol as a stabilizing agent [13]. This method includes a single step with only two reagents and is one of the most environment-friendly sulfation methods [13].

The antioxidant activity of a polysaccharide may also be increased via conjugation with another antioxidant molecule. Polyphenols have been among the preferred molecules for such modifications in the last decade, largely due to their excellent antioxidant properties [18]. Among them, gallic acid is the most used polyphenol [19]. An environment-friendly method for conjugating polysaccharides to gallic acid was proposed by Curcio et al. (2009) [20]. In this method, vitamin C (ascorbic acid) is oxidized by hydrogen peroxide, which results in the formation of ascorbate and hydroxyl radicals. Then, hydroxyl radicals react with the polysaccharide, leading to the formation of macroradicals. These macroradicals are polysaccharides with unpaired electron regions that act as entry points for the phenolic compounds. Upon addition of gallic acid, carboxyl groups of gallic acid react with these entry points, and covalent bonds between the polysaccharide and gallic acid are formed.

The binding of gallic acid to a polysaccharide also can increase its utility as an antioxidant in the human body. This phenolic compound has a variety of promising therapeutic and industrial applications [21]. However, only a small amount of this gallic acid acts as an antioxidant in the human body due to its low absorption in the digestive system and intense first-pass metabolism through the liver [22]. The conjugation of gallic acid into polysaccharide molecules can facilitate the absorption of gallic acid by the digestive system due to the greater retention of amphiphilic conjugates in the gastrointestinal tract. Moreover, these conjugated polysaccharides may be metabolized in the liver upon absorption, and thereby lead to the formation of derivatives with antioxidant activity [21,23].

Dielectric barrier discharge (DBD) is another environment-friendly chemical modification method [14,24]. This method involves the use of plasma (a partially ionized gas containing charged particles) as a modifying agent for the studied sample. The DBD method allows the modification of materials at room temperature and does not require high temperatures. The method also offers the advantage of only a slight change in the size of the materials [15]. In the DBD process, several reactive species are produced, which can promote oxygen insertion into molecules [25]. The sample also becomes acidified after a few hours of treatment [26] and may later acquire new conformations upon hydration. Such structural changes in biomolecules may be important for biological activities.

Most laminarins studied so far have been obtained from seaweeds growing in cold climate regions. On the other hand, data on algal laminarins from tropical regions is limited. The tropical brown seaweed *Lobophora variegata* has been previously investigated by our research group with respect to the production of sulfated polysaccharides [26] with antioxidant activities and possible cytotoxic effects [27]. However, laminarin production by these algae has not yet been investigated. Moreover, studies on the chemical modification of laminarins are limited, and DBD carboxylation and gallic acid conjugation of laminarins were not demonstrated before.

The aim of this study was thus to investigate the in vitro antioxidant activity of native laminarin extracted from brown seaweed *Lobophora variegata* and the effect of chemical modification methods on this activity. Three environment-friendly “green” modification methods, the DBD method, conjugation with gallic acid, and sulfation were investigated. Moreover, cell-protective effects of the laminarin with the best antioxidant profile (gallic acid conjugate) was evaluated against different conditions of oxidative stress caused by hydrogen peroxide.

## 2. Materials and Methods

### 2.1. Materials

We obtained acetonitrile, iron (II) sulfate and potassium ferricianyde (III), and sulfuric acid from Merck (Darmstadt, Germany). Moreover, ammonium molybdate, ascorbic acid, dextran standards, Folin–Ciocalteu reagent, gallic acid, hydrogen peroxide, methionine, nitro blue tetrazolium (NBT), riboflavin, sodium acetate, and sodium phosphate were obtained from Sigma-Aldrich Co. (St. Louis, MO, USA). Other reagents used in this study were of analytical grade.

### 2.2. Extraction of Laminarin

The brown seaweed *Lobophora variegata* was collected from Pirangi beach (Rio Grande do Norte, Brasil, 6° 00′ 50.7″ S/35° 10′ 83.2″ W). The *L. variegata* was washed, dried, crushed, and stored at the Laboratory of Biotechnology of Natural Polymers (BIOPOL, Natal, RN, Brazil), Department of Biochemistry, at Federal University of Rio Grande do Norte—UFRN. The seaweed was identified according to its morphology [28] and a voucher specimen was deposited in the UFRN Herbarium of the Institute of Biosciences, Federal University of Rio Grande do Norte, under the registration code UFRN 25510. The material collection occurred under the authorization of the Brazilian National Management System Genetic Heritage and Associated Traditional Knowledge (loose translation) SISGEN no A0D4240.

The algal polysaccharides were extracted, fractionated, and the fractions were named F0.3; F0.5; F0.8; F1.0; F1.5; and F2.0 as previously described [27]. The two laminarin-rich fractions (F0.8 and F1.0) were pooled, diluted with water (10 mg/mL), and fractionated using Amicon^®^ Ultra centrifugal filters (Merck-Millipore, Burlington, MA, USA). The use of these filters was associated with centrifugation (4500× *g*/20 min). Laminarin was obtained from material that passed the 30 kDa cut-off filter and was retained in the 10 kDa cut-off filter (i.e., laminarin has a size smaller than 30 kDa and greater than 10 kDa). Subsequently, the material removed from the filter with distilled water, lyophilized, and stored in closed bottles at room temperature, protected from light until use.

### 2.3. Laminarins Modification

#### 2.3.1. Laminarin Sulfation

Laminarin sulfation of *L. variegata* algae was performed as described in Telles et al. [13]. After the sulfation process, the sulfated laminarin was suspended in distilled water, lyophilized, and stored in closed bottles at room temperature, protected from light until use.

#### 2.3.2. Conjugation of Laminarin with Gallic Acid

Conjugation of laminarin with gallic acid was done as previously described [4]. Briefly, 50 mg of laminarin was dissolved in 5 mL of distilled water (final concentration 10 mg/mL). Then, 100 µL H_2_O_2_ (1.2%) and 5.4 mg ascorbic acid were added to the solution. The solution was incubated at room temperature for 30 min, protected from light. Subsequently, to this solution was added gallic acid (21.87 mg) in a ratio of 1 mol of gallic acid to each 1 mol of laminarin repeating units (one glucose dimer was considered as laminarin repeating unit), and the reaction was incubated at room temperature (22 °C) for 24 h, protected from light. The solution was then centrifuged using Millipore’s Amicon ^®^ Ultra-15 centrifugal filter with 3 kDa cut-off until all unreacted gallic acid was removed. The gallic acid conjugated laminarin solution was frozen and lyophilized for later use.

#### 2.3.3. Dielectric Barrier Discharge Modification

Dielectric barrier discharge (DBD) modification occurs through the application of dielectric barrier discharge [29,30]. The apparatus used for the generation of DBD plasma is composed of the gas inlet, tube (where plasma is generated), metal plate (where the sample is deposited), and a dielectric barrier, which simultaneously isolates the system and spreads the plasma, distributing it throughout the surface of the plate and consequently on the sample and the spot where the discharge is applied. Laminarin (10 mg) was placed on the metal plate and was subjected to a plasma generated from a continuous flow of helium (1 L/min) into the device and exposure of this helium gas to a voltage of 15 kV at 4 kHz for 1 min on discharge.

### 2.4. Physical and Chemical Characterization of Samples

#### 2.4.1. X-ray Dispersion Analysis

Elemental analysis of the laminarins was carried out using an X-ray dispersion spectrometer (MEV-HITACHI Bruker brand Auriga model XFlash Detector) located in the Laboratório de Microscopia Eletrônica de Varredura (LABMEV) at Materials Engineering Department (DEMat), the Federal University of Rio Grande do Norte (UFRN). The acquisition time was 40.0 s, and the process time was 5 s. The accelerating voltage was 15.0 kV. At least three analyses were performed in different fields.

#### 2.4.2. Determination of Protein, Sulfate, Uronic Acids, Phenolic Compounds Content, and Monosaccharide Composition

Sulfate content was measured after acid hydrolysis (HCl 6 N, 6 h, 100 °C) using the turbidimetric method as described earlier [31]. Protein content was quantified with Coomassie Brilliant Blue reagent and bovine serum albumin as standard as described in Reference [32]. Phenolic compounds were measured by the Folin–Ciocalteau reagent as described in Reference [13]. Carboxyl groups were quantified using the uronic acid assay using carbazole reagent [33]. Monosaccharide composition was quantified as described in Reference [13].

#### 2.4.3. Agarose Gel Electrophoresis in 1,3-diamino Propane Acetate Buffer (PDA)

The electrophoretic mobility of laminarins was evaluated by gel electrophoresis in PDA buffer, as described earlier [31]. Initially, glass slides were coated with 0.6% (*m*/*v*) agarose in PDA buffer (0.05 M, pH 9.0). Subsequently, aliquots of the laminarins and modified laminarins (about 50 µg) were applied to the gel and subjected to electrophoresis (100 V, 4 °C) for 60 min. After the electrophoretic run, the polysaccharides were precipitated with 0.1% cetyltrimethylammonium bromide (CETAVLON, Sigma Chemical Company, St. Louis, MO, USA) for 3 h at room temperature and the gels were dried using warm air stream. To visualize the samples, gels were stained with a solution of 0.1% toluidine blue in 1% acetic acid and 50% ethanol. The gel was then de-stained with the same solution without the dye. Fucan A (a sulfated polysaccharide) was kindly donated by M.Sc. Monica O. R. Amorim (Department Biochemistry, UFRN, Natal, RN, Brazil).

#### 2.4.4. Determination of Laminarins Molecular Weight

The apparent molecular weight of samples was determined by high-performance size exclusion chromatography (HPSEC) (GE Healthcare Bio-Sciences, Pittsburgh, PA, USA) on TSK-Gel^®^ 3000 (30 123 cm × 0.75 cm), with a column temperature of 30 °C. The samples were eluted with ultrapure water and 0.1 M NaNO2 acetate buffer, at a flow rate of 0.6 mL/min and detected by a refractive index detector. The column was calibrated using different dextrans (10; 47; 74; 147 kDa) purchased from Sigma (St. Louis, MO, USA).

### 2.5. In Vitro Antioxidant Tests

Six in vitro antioxidant tests were used, total antioxidant capacity, superoxide radical scavenging assay, hydroxyl radical scavenging assay, ferrous chelating assay, cupric chelating assay, and total antioxidant capacity (TAC). All of them were carried out as described by Presa and co-workers [34]. The sixth method, reducing power, was carried out according to the method described by Galinari and co-workers [35].

#### 2.5.1. Determination of Total Antioxidant Capacity (TAC)

Briefly, the solution (1 mL) containing the samples (0.1 mg/mL), ammonium molybdate (4 mM), sodium phosphate (28 mM), and sulfuric acid (0.6 M) were added into a tube, stirred, and incubated (100 °C, 90 min). The tubes were cooled and were read at 695 nm wavelength. The standard used was ascorbic acid (AA) and the results were expressed as AA equivalent per gram of sample.

#### 2.5.2. Superoxide Radical-Scavenging Assay

As described by Presa et al. [34], 1 mL of the samples (at different concentrations) were mixed with 100 mM ethylenediaminetetraacetic acid (EDTA), 50 mM phosphate buffer (pH 7.8), 13 mM methionine, 75 mM nitroblue tetrazolium (NBT), and 2 mM riboflavin to form a 3 mL solution. The entire reaction assembly was carried out in dark. After 10 min, the formazan forms were monitored at 560 nm. Identical tubes with distilled water and reaction mixture were used as a blank. Gallic acid was used as standard (from 0.01 to 0.6 mg/mL). The results were expressed according to the equation:

% of activity = ([Acontrol − Asample]/[Acontrol − Ablank]) × 100

where Acontrol: absorbance of the control tube, Asample: absorbance of the sample tube, and Ablank: absorbance of the blank tube.

#### 2.5.3. Hydroxyl Radical Scavenging Assay

The OH radical scavenging activity of samples was investigated using Fenton’s reaction (Fe^2+^ + H_2_O_2_ → Fe^3+^ + OH^−^ + OH). The data were expressed as the inhibition rate. For OH production, the samples (at different concentrations) were added to 3 mL sodium phosphate buffer (150 mM, pH 7.4), which contained 10 mM FeSO_4_.7H_2_O, 10 mM EDTA, 2 mM sodium salicylate, 30% H_2_O_2_. In the control, sodium phosphate buffer replaced H_2_O_2_. After 37 °C for 1 h, OH radical was detected by monitoring absorbance at 510 nm using a microplate reader. Gallic acid was used as a positive control.

#### 2.5.4. Iron-Chelating Assay

The samples at different concentrations were added to a solution containing FeCl_2_ (2 mM). After ferrozine (5 mM) was added to the mixture, the solution was mixed and kept for 10 min at 37 °C. Ultrapure water was used as blank and EDTA was used as standard. The samples were monitored at 562 nm using a microplate reader. The results are expressed in accordance with the equation:

% of chelation = ([Acontrol − Asample]/Acontrol) × 100

where Acontrol: absorbance of the control tube and Asample: absorbance of the sample tube.

#### 2.5.5. Copper-chelating Assay

The test was performed in 96-well microplates with a reaction mixture containing different concentrations of samples (0.1–20 mg/mL), copper II sulfate pentahydrate (50 mg/mL), and pyrocatechol violet (4 mM). All wells were homogenized with the aid of a micropipette, and the solution absorbance was measured at 632 nm. The ability of the samples in chelating the copper ion was calculated using the following equation:

(Absorbance of blank) − (Absorbance of the sample)/(Absorbance of the blank) × 100

#### 2.5.6. Reducing Power

Briefly, phosphate buffer (0.2 M, pH 6.6) containing potassium ferricyanide (1%) was mixed with samples in different concentrations (0.1–1 mg/mL) to final volume of 1.0 mL. After 20 min at 50 °C, the reaction was stopped with 10% trichloroacetic acid (TCA). One milliliter of Ferric chloride (0.1%) in distilled water was added to the mixture and the absorbance was measured at 700 nm. The results were expressed as percentage activity of 0.1 mg/mL ascorbic acid (standard), which corresponded to 100% activity.

### 2.6. Structural Characterization of Native Laminarin and Gallic Acid Conjugated Laminarin

#### 2.6.1. Fourier Transformed Infrared Spectroscopy (FT-IR)

Fourier transform infrared spectroscopy (FT-IR) analysis was performed by pressing 5 mg of each sample with 10 mg of KBr, and the resulting material was analyzed using a spectrometer Shimadzu FTIR-8400S (Kyoto, Japan). Thirty scans were performed for each sample at room temperature to obtained infrared spectra with a range between 400 and 4000 cm^−1^. The correction of the baseline was calculated using an IRSolution software version 1.60SU1 (Shimadzu, Kyoto, Japan).

#### 2.6.2. Nuclear Magnetic Resonance (NMR) Spectroscopy

Nuclear Magnetic Resonance (NMR) Spectroscopy analyses were performed dissolving the samples (20 mg) in 600 μL of deuterium oxide (D_2_O). All the NMR analyses were obtained in a Bruker Avance III 600 MHz spectrometer (Bruker BioSpin Corporation, 138 Billerica, MA, USA) equipped with a 5-mm inverse quadruple resonance probe (QXI) at 70 °C. ^1^H-NMR spectra were obtained using number of scans (NS) = 8, spectral width (SWH) of 6393.862 Hz, and size of fid (TD) = 64 k. 2D-NMR (^1^H/^13^C) HSQCed (Edited Heteronuclear Single Quantum Coherence) analysis were performed using Bruker’s hsqcedetgpsisp2.3 pulse sequence with NS = 32, number of dummy scans (DS) = 128, TD = 2048 (F2) × 200 (F1), SWH = 6393.862 Hz (F2) × 30182.674 Hz (F1), and relaxation delay = 1.0 s. The chemical shifts were expressed in δ relative to sodium trimethylsilyl propionate (TMSP) at δ = 0.00 in accordance with IUPAC recommendations.

### 2.7. Cell Culture Experiments

In order to test the protective or reparative capacity of laminarins against the oxidative damage on Madin–Darby canine kidney (MDCK) cells caused by H_2_O_2_, a method adapted from Ouyang et al. (2011) was performed as follows. First, optimum conditions for maximum cell damage were determined. MDCK dog kidney cell line (ATCC^®^ CCL-34 ™) was chosen for this purpose. Cells were placed in 96-well plates (4 × 10^3^ cells per well) containing Dulbecco’s Modified Eagle Medium(DMEM) without fetal bovine serum (FBS) and with different concentrations (0.1 to 5 mM) of hydrogen peroxide (H_2_O_2_) for 1 h. Afterward, the H_2_O_2_-containing medium was replaced with DMEM containing FBS. After 24 h of incubation, the 3-(4,5-dimethylthiazol-2-yl)-2,5-diphenyl-tetrazolium bromide colorimetric (MTT) reduction ability of cells was evaluated. Here, the presence of H_2_O_2_ (3 mM) caused the cells to suffer enough damage to decrease their ability to reduce MTT by 50% (data not shown).

Two experimental settings were used to test the antioxidative capacity of the samples on cells subjected to H_2_O_2_ stress [36]. In the first setting, the cells were first exposed to H_2_O_2_ (3 mM) followed by the addition of the polysaccharides. This setting was thus denoted as the “reparative effect assay”. In the second setting, H_2_O_2_ (3 mM) and polysaccharides were added to the medium concomitantly to test the protective ability of polysaccharides against oxidative stress, hence this setting was denoted as the “protective effect assay”.

For the reparative effect assay, cells were first starved for 24 h and then incubated in a serum-free medium containing H_2_O_2_ (3 mM). Negative control was also included, which was incubated in a medium without serum and H_2_O_2_. After 1 h of exposure to H_2_O_2_, the medium was replaced with serum medium containing the gallic acid-laminarin conjugate (LMG) at different concentrations (125, 250, and 500 µg/mL). After 24 h of incubation, cell viability was determined by using the 3-(4,5-dimethylthiazol-2-yl)-2,5-diphenyl-tetrazolium bromide colorimetric (MTT) test [37]. DMEM with H_2_O_2_ (3 mM) and with serum were used for positive and negative controls, respectively.

For the protective effect assay, cells were first incubated in a serum-free medium for 24 h. The medium was then exchanged with serum-containing LMG at different concentrations (125, 250, and 500 µg/mL) and 3 mM H_2_O_2_. The cells were further incubated for 2 h in this medium. Afterward, the medium was exchanged with serum, and cells were incubated for another 24 h. Finally, cell viability was determined by using the MTT test [37]. DMEM with H_2_O_2_ (3 mM) and with serum were used for positive and negative controls, respectively.

#### 2.7.1. Annexin V-FITC/PI Double Staining and Analysis by Flow Cytometry

FITC/Annexin V Apoptosis Kit was used to evaluate the effects of samples on cell death/survival. Cells were grown in 6-well plates until they reached a confluence of 2 × 10^5^ cells/mL when they were stimulated to enter G0 in a medium without serum for 24 h. Next, cells were to exit G0 by adding DMEM medium supplemented 10% de FBS, in the presence of samples (0.125 mg/mL).

After 24 h, the cells were harvested, collected, and washed with cold phosphate-buffered saline (PBS). The supernatant was discarded, and cells were resuspended in 200 µL of 1x binding buffer. 5 µL of Annexin V-FITC and 1 µL of PI solution 100 µg/mL was added in 100 µL of cell suspension. Cells were incubated for 15 min at room temperature and kept under light protection. Then, these cells were analyzed by flow cytometry the incubation time, 400 µL of binding buffer for annexin V (1x) (flow cytometer FACSCanto II, BD Biosciences Eugene, OR, EUA), software FACSDiva, version 6.1.2 (Becton Dickson, Franklin Lakes, NJ, EUA). Measuring fluorescence emission at 530–575 nm for annexin V and 630/22 nm for PI. FlowJo software v. X10.0.7, 2015 (Tree Star, Inc., Ashland, OR, USA) was used for data analysis. Negative control was prepared without the presence of samples, and a gallic acid control was prepared with a gallic acid content from LMG.

#### 2.7.2. Cell Cycle

2 × 10^5^ cells and six-well plates were incubated for 24 h with serum-free medium. Then, the cell medium was exchanged with serum containing the samples (LM and LMG) at a concentration of 125 µg/mL, and the cells were incubated for 24 h. Subsequently, the serum medium and samples were removed, cells were trypsinized (500 µL/well), washed with PBS, and centrifuged at 3200 rpm for 5 min at 4 °C. The cells were fixed with 70% ethyl alcohol and incubated for 60 min at 4 °C. Then, the cell membrane was permeabilized with 90 µL triton X, and RNAse (4 mg/mL, 37 °C for 60 min) was added. Ten microliters propidium iodide (1 mg/mL) and 200 µL PBS were added. Reading was performed on the cytometer (FACSCanto II, BD Biosciences Eugene, OR, USA), FACSDiva software, version 6.1.2 (Becton Dickson, Franklin Lakes, NJ, USA).

### 2.8. Statistical Analysis

All data are presented as the mean ± standard deviation (*n* = 3). The ANOVA test was performed to check the difference between results. The Student–Newman–Keuls test (*p* < 0.05) was used to solve similarities found by ANOVA. All tests were performed in GraphPad Prism 5 (GraphPad Softwares, San Diego, CA, USA).

## 3. Results and Discussion

After performing the chemical modifications described in the Methods section, three types of laminarins were obtained: Sulfated laminarin (LMS), DBD-modified laminarin (LMC), and gallic acid-laminarin conjugate (LMG). These compounds were evaluated in different tests along with the native (unmodified) laminarin (LM). The results of our evaluation are described below.

### 3.1. Physicochemical Characterization

#### 3.1.1. Determination of the Apparent Molecular Weight (MW) of Laminarins

The apparent molecular mass (MW) of polymers is an important characteristic that can influence biological polymer activities. The MW of the native laminarin was first determined using HPLC and then compared to the MWs of the modified laminarins. The MW of native LM was found to range between 10 and 14 kDa, with most of the LM molecules showing an MW of around 12.4 kDa. Modified laminarins showed a narrower MW distribution than that of the native LM, and the MWs of these laminarins were highly similar to each other, which were found to be 12.8, 12.6, and 12.8 kDa for LMS, LMC, and LMG, respectively.

Generally, chemical modifications, such as the addition of functional groups to the structure of a polysaccharide, lead to peaks with larger bases in HPLC analyzes. This occurs due to the polydispersion characteristic of the polysaccharides, which means that the modifications in each of the molecules existing in the polysaccharide sample do not occur to the same extent. Therefore, it leads to the formation of a population of molecules more heterogeneous in relation to molecular mass compared to the native sample. However, this fact was not observed here, the peaks obtained with the modified laminarins were narrower than that obtained with the native laminarins. A possible explanation for this is that with the chemical modifications, the modified laminarin molecules have assumed more similar conformations and, consequently, they became less polydisperse, which led to peaks with narrower bases.

The sulfation of LM led to only small changes in MW. The decrease in the MW following the sulfation process was previously observed following the sulfation of other β-glucans [38,39,40], indicating degradation. This degradation was attributed to sulfation conditions. The authors of these studies studied 1665 kDa glucans and obtained sulfated glucans with MWs ranging between 20.9 and 484.8 kDa only by modifying the duration of the sulfation reaction. Biological/pharmacological activities of sulfated laminarins were investigated in dozens of studies, yet only a few reported structural characterization results [41]. In one such study, Ménard and co-workers [42] adapted Alban’s method and obtained sulfated but undegraded laminarins. The similarity between our sulfation method here and the Alban and Ménard method may explain why our sulfation process did not cause degradation of laminarins.

To the best of our knowledge, conjugation with gallic acid or DBD modification of laminarins was not demonstrated before. A comparison of our results with previous studies was, therefore, not possible. Nevertheless, our results show that these modifications did not change the MW compared to that of native LM. The application of the gallic acid conjugation method used here on another polysaccharide, chitosan, was shown to result in a reduction of 10% [4] to 25% of chitosan MW [43]. Chitosan molecules are linear polymers and, therefore, more susceptible to degradation by hydrogen peroxide action. Regarding applications of glucans, the conjugation of gallic acid with dextran (α-glucan) was reported in just a single study previously [11], where a 25% loss in MW was observed after the procedure. Like laminarin, dextran is a branched polymer. However, dextran molecules are α-(1-4)-glucans, and α bonds in their structure are more susceptible to breakage than beta bonds. This may explain the MW loss for dextran compared to laminarin.

#### 3.1.2. X-ray Dispersion Analysis

All samples were found to show polysaccharide characteristics, as they included carbon, oxygen, and hydrogen in similar quantities. Elemental compositions of laminarins also did not change in LMC and LMG, indicating that the chemical modifications by DBD and conjugation with gallic acid did not lead to the insertion of additional elements other than C, O, and H, such as nitrogen. The sulfur content was also only observed for LMS, indicating the successful sulfation of this polymer.

#### 3.1.3. Electrophoretic Profile of the Samples

**Figure 1:**
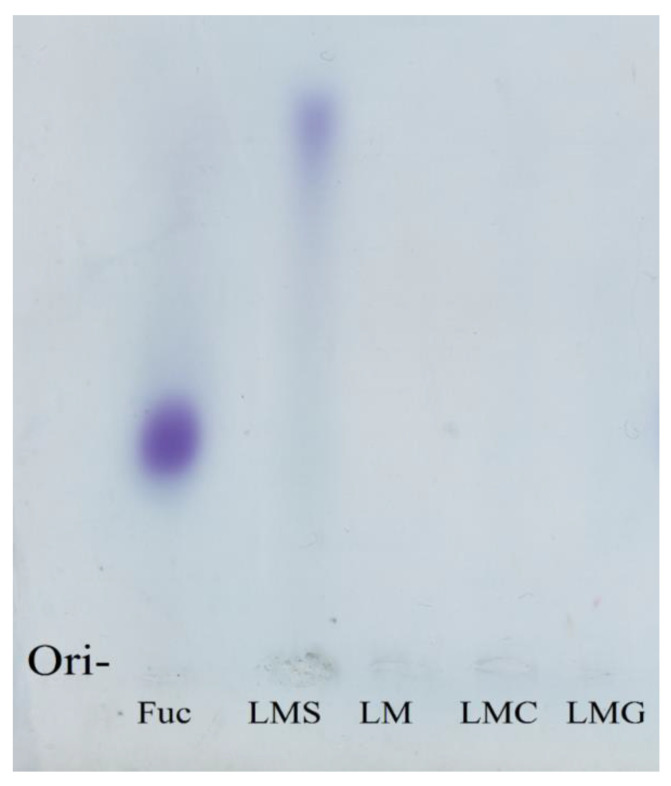
Electrophoretic profile of Fucan A (Fuc) from *Spatoglossum schöederi* seaweed; LMS: sulfated laminarin; LM: native laminarin; LMC: carboxylated laminarin; LMG: gallic acid–laminarin conjugate. Fifty micrograms of each sample were subjected to electrophoresis.

Sulfated polysaccharides react with toluidine blue due to the presence of sulfate groups and yield a purple color (metachromasia) [17]. For this reason, we used a fucan, a natural sulfated polysaccharide, as a positive control, which in turn indicated the color that sulfated polysaccharide should have when exposed to toluidine blue. In Figure 1 the fucan yielded purple color in electrophoresis. In line with its sulfate content, LMS was also the only laminarin yielding purple color here.

#### 3.1.4. Determination of Protein, Sulfate, Uronic Acid, Glucose, and Phenolic Compound Content

Protein, sulfate, uronic acids, and phenolic compound contents of the samples were determined (Table 1). The proportions of the monosaccharides in each sample are also given in Table 1.

A small proportion of LM content was detected to include proteins (~0.2%). No protein was detected upon application of LMS and LMC methods. The protein quantification method used here is based on the binding of the dye molecule to aromatic amino acids of the proteins [32]. Since gallic acid is an aromatic molecule, dye molecules were possibly associated with some of the gallic acids within the LMG, and therefore the protein content detected in the LMG sample (~0.4%) is possibly a false positive.

The identification of sulfate in only LMS further proves successful sulfation of LM. Here, the sulfate content was found to be approximately 1.3%, whereas commercial sulfated laminarins include around 15.3% sulfate [44]. In another glucan, sulfate was observed at a ratio of 5.8% [45]. The discrepancy between our results and those reported in previous studies may be explained by the structural characteristics of the laminarin produced by *L. variegata*. All laminarins show the characteristics of β-glucans, yet the structures of laminarins from different sources may differ from each other [46]. Properties such as size, type of branches, degree of branching, and solubility may influence the susceptibility of laminarins to the addition of the substituent groups such as the sulfate group [47]. The level of branching in laminarins is positively correlated with the degree of sulfate substitution. Low levels of sulfation in LM upon modification may indicate fewer branches compared to the laminarins including 15.3% sulfate [44]. Hence, the low degree of sulfation may be related to the structural properties of laminarin produced by *L. variegata*.

On the other hand, carboxyl groups (11.7%) were found exclusively in LMC. This indicates that the DBD modification carboxylated LM. To the best of our knowledge, there is no previous report on the carboxylation of laminarins or other algal polymers either via DBD modification or using any other method. However, carboxymethylation of laminarins was demonstrated before, and in these studies, the degree of substitution was much lower than that observed here [48,49,50].

Phenolic compounds were not detected in LM, LMS, and LMC. On the other hand, LMG included a small amount of phenolic compounds (~1.5%). This result may be explained by the presence of aromatic gallic acid molecules bound to laminarin. The level of phenolics found here was higher than those observed by Curcio et al. [20] and Queiroz et al. [4], which were 0.7% and 1.0%, respectively. However, it is also worth noting that these authors worked with chitosan, a linear polysaccharide.

Regarding the monosaccharide composition, LM, LMS, and LMG have only glucose. This indicates that the laminarin separation process using the filtration devices was efficient and that LM and LM modifications were homoglucan. The presence of glucuronic acid in LMC was due to the carboxylation processes.

### 3.2. In Vitro Antioxidant Activity

#### 3.2.1. Superoxide and Radical Hydroxyl Scavenging Activities

β-Glucans such as laminarins have a low hydroxyl and superoxide radical scavenging capacity [9]. For example, laminarin extracted from *Cystoseira barbata* did not show any hydroxyl radical scavenging activity even at high concentrations (2 mg/mL) [10]. On the other hand, there are also exceptions, such as the β-glucan extracted from *Botryosphaeria rhodinan* which displayed good scavenging activity against hydroxyl radicals (~90%) and superoxide anions (~40%) at 3 mg/mL concentration [51]. Radical scavenging capacities of the β-glucan may thus vary depending on the structural characteristics of the glucans. Therefore, radical scavenging activities of novel β-glucans, including laminarins, should be evaluated. Hence, laminarins studied here were evaluated with respect to their radical-scavenging capacities. None of the samples showed any scavenging activity even at the highest concentration evaluated (1000 µg/mL).

Hydroxyl or superoxide scavenging activities of sulfated or carboxylated laminarins have not been evaluated before. However, sulfation was found to increase the scavenging activity of glucans in previous studies on sulfated β-glucans [52,53,54]. On the other hand, these results should be interpreted with caution, as the scavenging capacity of native glucans was evaluated at the same concentration (m/v, for example, mg/mL) as their sulfated derivatives. However, the same authors also showed that sulfation leads to a decrease in molecular weight. This decrease can be very drastic such as that shown by Wang and co-workers (2012). Sulfation of a glucan was also shown to decrease the molecular weight from 25.18 × 10^4^ kDa to 1.684 × 10^4^ kDa [39]. Therefore, it is also possible that the increase in radical scavenging activity following sulfation may not be due to a direct effect of sulfation but due to methodological flaws in these studies.

Carboxylation of other polysaccharides was previously observed to increase hydroxyl radical scavenging activities, yet this method also causes degradation of the native polymer [52,53]. The effectiveness of carboxylation in increasing the scavenging activity is controversial, mainly due to the reasons explained above.

Altogether, the data shown here indicate that the modifications on LM did not enhance its action as a radical scavenging agent. Another strategy to reduce the harmful effects of hydroxyl radicals is through the chelating action of metals [55]. This is achieved through the participation of metal ions in the generation processes of these radicals [56]. Iron and copper ions are presented in large quantities in organisms, and the reduction of their oxidative effects is of great relevance for the normal functioning of cells [57]. Therefore, iron- and copper-chelating capacities of the samples were evaluated as well.

#### 3.2.2. Iron- and Copper-chelating Activities

Neither native LM nor modified LMs showed any iron-chelating activity under any of the evaluated concentrations (0.1–2.0 mg/mL). In contrast, the laminarin from *Cystoseira barbata* was shown to have a free iron ion chelating capacity of 78% at a concentration of 20 mg/mL [10]. These results corroborate the fact that the antioxidant activity of glucans, as well as other polysaccharides, depends on their structural characteristics.

**Figure 2:**
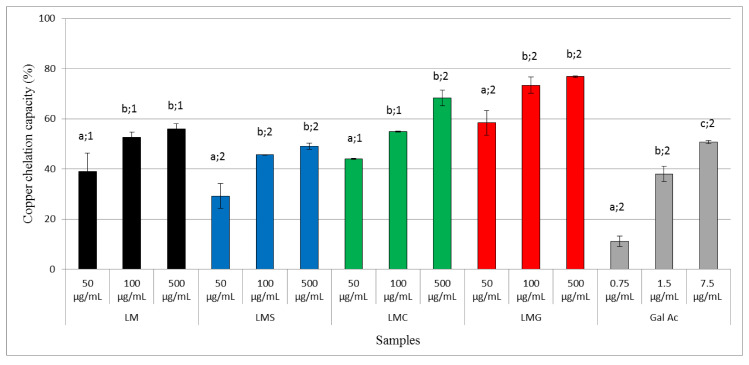
Copper-chelating activities of 50 µg/mL, 100 µg/mL, and 500 µg/mL LM (**black**), LMS (**blue**), LMC (**green**), and LMG (**red**). The gallic acid (Gal Ac) standard (in **gray**) was used at the amounts corresponding to the used LMG concentrations. Different letters represent significant differences between LM and LMS/LMC/LMG at different concentrations. Different numbers represent a significant difference between LM and LMS or between LM and LMC or between LM and LMG at the same concentrations (*p* < 0.05).

Figure 2 shows that LM (100 µg/mL) yielded a maximum copper-chelating activity of 50%, and this activity did not change as concentration was increased (500 µg/mL). LMS also showed copper-chelating activity. However, the activity of LMS was lower than that of the native LM at all evaluated concentrations.

**Figure 3:**
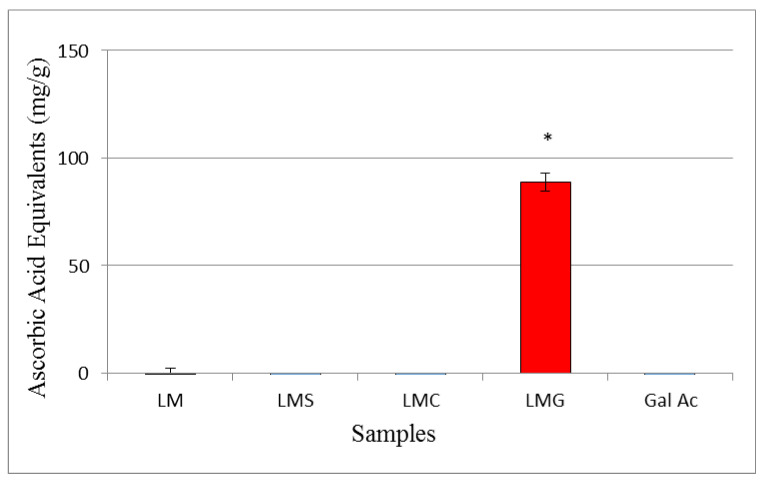
Total antioxidant activities of 100 µg LM (**black**), LMS (**blue**), LMC (**green**), LMG (**red**), and gallic acid (Gal Ac.). The asterisk (*) represents a significant difference between LM and another sample (*p* < 0.05).

In contrast to sulfation, carboxylation of LM led to the formation of LMC with a good copper-chelating activity. As shown in Figure 3, LMC (500 µg/mL) was able to chelate about 70% of the copper ions in the solution. Nonetheless, the highest chelating activities were obtained for LMG (500 µg/mL) with values reaching as high as 80%. This result is in contrast to that observed with chitosan: conjugation of chitosan with gallic acid did not increase the chelating activities [4].

#### 3.2.3. Total Antioxidant Activities

Using the total antioxidant capacity test (TAC), the capacity of the samples to donate electrons in an acid medium was determined. The results are presented in Figure 3. Here, LMG was the sample that showed the highest activity with approximately 89 mg of ascorbic acid equivalent.

#### 3.2.4. Reducing Power

**Figure 4:**
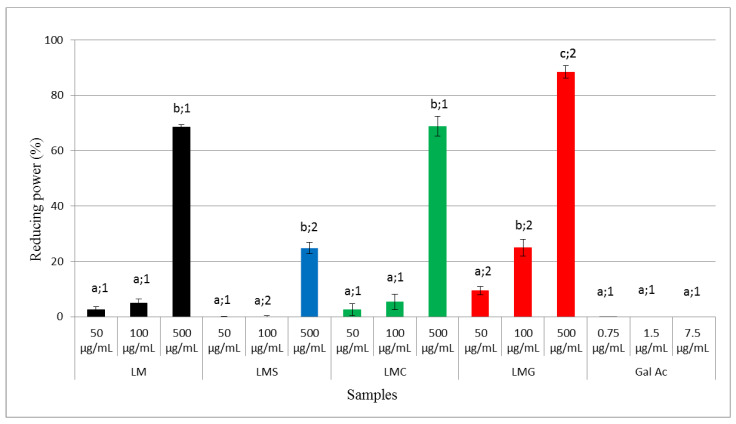
Reducing powers of LM (**black**), LMS (**blue**), LMC (**green**), and LMG (**red**). The gallic acid (Gal Ac) was used at the amounts corresponding to the used LMG concentrations. Different letters represent significant differences between LM and LMS/LMC/LMG at different concentrations. Different numbers represent a significant difference between LM and LMS or between LM and LMC or between LM and LMG at the same concentrations (*p* < 0.05).

Using the reducing power test, the capacity of the samples to donate electrons in an environment with pH near neutral was determined. LM (500 µg/mL) was found to show a reducing power of 80% (Figure 4). This reducing power was not modified by carboxylation and decreased only after the sulfation process. Otherwise, the LMG obtained a higher activity than the LM at all evaluated concentrations (Figure 4).

The decrease in the reducing power activity of a sulfated polysaccharide has been previously reported for the native polysaccharide of *Ganoderma atrum* [58]. The decrease in the number of hydroxyl groups and the change in conformation of the polysaccharide caused by the addition of sulfate groups were proposed to decrease the hydrogen donation ability of the molecule. This may explain the lower activity of LMS compared to LM.

LMG showed higher reducing power than LM at all tested concentrations. In addition, it was the unique compound to shown activity in the TAC test. The ability of the chitosan to donate electrons under different chemical conditions, TAC and reducing power tests, was also shown to increase after conjugation with gallic acid [4,59]. However, unlike chitosan conjugated to gallic acid, LMG presented a dramatic increase in the TAC.

Several authors proposed that the increase in reducing power is unrelated to the activity of the gallic acid alone, but rather due to the higher reducing power of the resulting conjugate [4,11,59]. In fact, LM showed a low electron-donating capacity, as well as gallic acid has a low electron-donating capacity at the concentrations in which it is found in LMG (500 µg of LMG contains 7.5 µg of gallic acid). These data (Figure 3 and Figure 4) do not indicate a simple joint action of the molecules (acid gallic and laminarin) as reducing agents and that probably the conjugation of these molecules made a molecule (LMG) more reactive. These corroborate the data presented by Queiroz et al. [11], who combined gallic acid and dextran and obtained a molecule with greater reducing power than the sum of gallic acid and dextran activities.

Therefore, Wang et al. [60] proposed that the antioxidant activity is not only related to the addition of functional groups but also to the structural conformation of the polysaccharide after modifications. What can explain LMG being so effective as an electron donor in the TAC test? Both TAC and reducing power tests were used in the present study to evaluate the ability of the sample to donate electrons under different chemical conditions, acidic pH (TAC test), and neutral pH (reducing power test). Therefore, we suggested that the acidic pH creates an environment that induces a LMG structural conformation more able to donate electrons and act as a reducing agent than neutral pH. However, to figure out the mechanism by which the presence of many hydrogen ions enhance the antioxidant action of LMG is beyond the objectives of this paper, and future studies are warranted to evaluate these characteristics to determine the correlation between the structure and antioxidant activity of LMG.

In general, LMG was shown to present the highest antioxidant activity in vitro and, therefore, included in further structural characterization and antioxidant activity tests on cells.

### 3.3. Structural Characterization

#### 3.3.1. FT-IR Analysis

**Figure 5:**
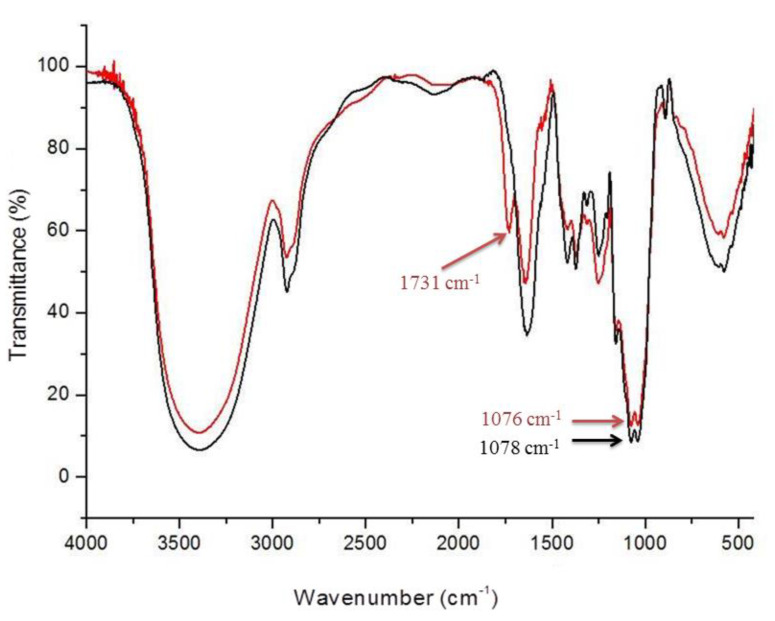
Infrared spectra of LM (in black) and LMG (red).

The FT-IR spectrum allows for rapid identification of functional groups present in a molecule. Figure 5 shows the infrared spectra of LM and LMG, and Table 2 shows the main bands present in Figure 5. LM (black) and LMG (red) showed similar profiles, as evidenced by the presence of bands at 3400, 3000, 1400, 1100, and 1000 cm^−1^ on both spectra, which represent vibrations of hydroxyl group, CH, asymmetric C=O, C-O-O, and C-O [61,62], respectively. The presence of organic components within the structures was also confirmed. The C-O-C vibration at 1100 cm^−1^ and the C-O stretch at 1000 cm^−1^ are indicators of the presence of glyosidic bonds [63,64], and hence polysaccharides. Another interesting band was found at 890 cm^−1^, indicating β-configuration of the monosaccharides [65].

The presence of gallic acid in LMG was also confirmed by the band at 1737 cm^−1^, which only occurs in the LMG spectra and indicates the presence of carbonyl esters formed by the bond between the hydroxyl on a sugar and the carboxyl of gallic acid [63]. Other bands that could indicate the presence of gallic acid are the ones around 1537 and 1643 cm^−1^, which indicate the C–C and C=C vibrations on the aromatic rings, respectively. Nonetheless, these bands overlapped with other bands and, therefore, were not assigned.

#### 3.3.2. NMR Analysis

**Figure 6:**
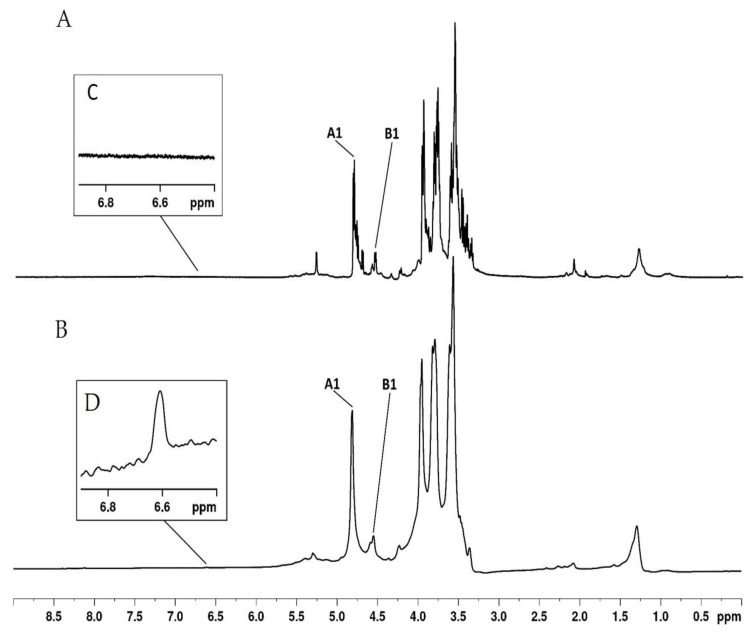
NMR spectra of LM (**A**) and LMG (**B**). The identified signals correspond to the typical signs of laminarins present in both samples. In (**C**) we highlight the region between 6.5 and 6.8 ppm in LM spectrum, where there is no signal. The signal at 6.60 ppm (**D**) is present only in the LMG spectrum and corresponds to the hydrogen from the aromatic ring of gallic acid.

**Figure 7:**
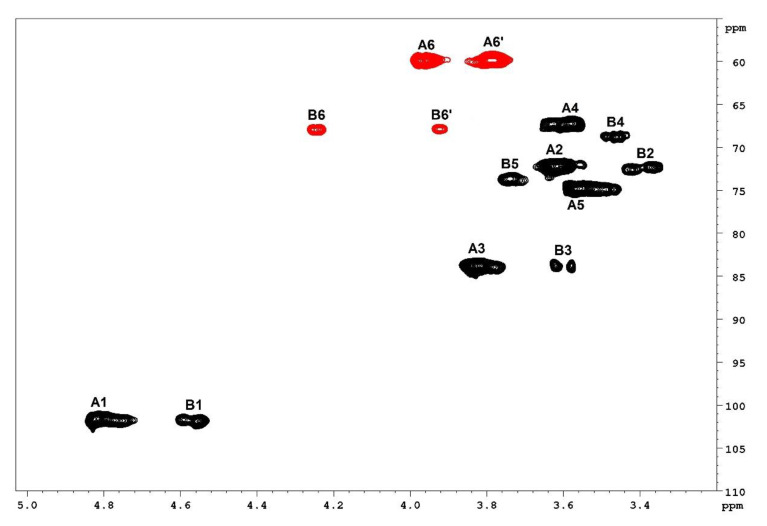
2D-NMR (HSQCed) spectrum of the LM. Letters refer to spin systems and numbers refer to the positions of spin systems.

The ^1^H-NMR spectra (Figure 6) of LM and LMG included no distinguishable anomeric signals, which is typical for polysaccharide spectra. However, an anomeric region from δ 4.50 to 5.00 ppm, which represents the characteristic region of β-anomers, was also determined. This anomeric region indicates that the samples contain two different units. The ^1^H-NMR spectra of both samples were remarkably similar to each other. This shows that the conjugation process did not introduce major changes in laminarin structure. Therefore, we performed a more detailed structural analysis of LM by two-dimensional NMR spectroscopy (2D-NMR). Two spin systems are evident from the HSQCed spectrum of LM (Figure 7), named units A and B, with anomeric hydrogen signals at δ 4.81 and 4.55 ppm, respectively. The corresponding chemical shifts were determined by analysis of the HSQCed spectrum (Table 3). Unit A is →3)-β-D-glucopyranose-(1→; unit B is →3,6)-β-D- glucopyranose-(1→.

In general, the spectra of LM and LMG were consistent with β(1→3)-glucan with few (1→6)-substituted glucosyl side chains like other laminarins [66,67,68,69].

The LMG spectrum included a signal in the region of 6.6 ppm, which was excluded from this spectrum. This signal has been identified for gallic acid–chitosan [4] and gallic acid–dextran [12] conjugates previously and corresponds to H of the aromatic ring [70]. Hence, the conjugation of gallic acid and laminarin was confirmed.

### 3.4. Evaluation of the Protective and Reparative Effects of LMG against Oxidative Stress on MDCK Cells

The protective and reparative effects of laminarins against oxidative stress caused by hydrogen peroxide (H_2_O_2_) were evaluated as well. Here, the presence of permanent oxidative stress conditions in vivo, as well as the presence of distinct groups of cells that were already or are not yet exposed to oxidative stress were considered, and two different experimental settings were used accordingly. The first settings were called the “reparative effect assay” and included the exposure of cells first to H_2_O_2_ and later to polysaccharides. The idea here was to determine whether the presence of polysaccharides can stimulate the regeneration of damaged cells. In the second setting (protective effect assay), the cells were exposed to H_2_O_2_ and the polysaccharides concomitantly. Here, it was evaluated whether polysaccharides can directly protect cells from damage caused by the H_2_O_2_.

#### 3.4.1. Reparative Effect Assay Results

Polysaccharides from green tea (at concentrations between 20 and 100 µg/mL) were shown to act in a reparative manner against oxidative damage on HK-2 (human kidney proximal tubular epithelial) cells [71]. These cells were exposed to the polysaccharides and an agent that induces oxidative damage, and later showed the viability of approximately 90%, whereas the viability of cells exposed to oxidative damage remained around 60%. Protective effects of the LMG was, therefore, evaluated here as well.

**Figure 8:**
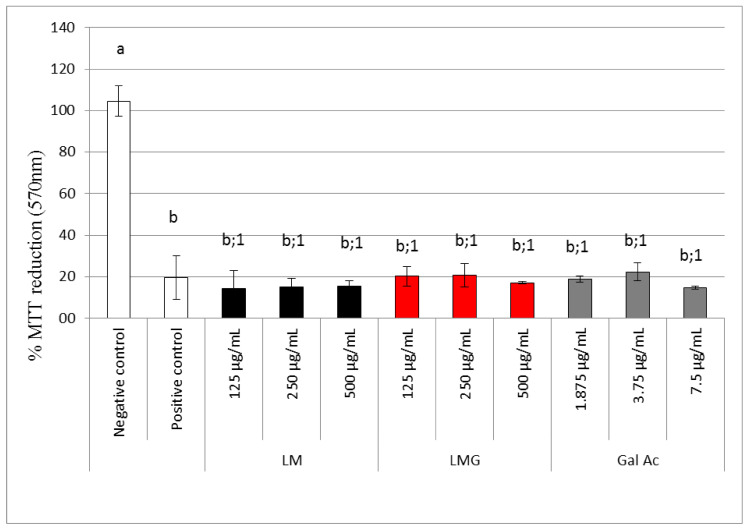
The 3-(4,5-dimethylthiazol-2-yl)-2,5-diphenyl-tetrazolium bromide colorimetric (MTT)-reducing activity of Madin–Darby canine kidney (MDCK) cells treated with H_2_O_2_ and subsequently with LMG for 24 h (Repairing effect). Values are expressed as mean ± standard deviation. Different numbers represent significant differences between controls and LMG (*p* < 0.05). Gal Ac – gallic acid.

Figure 8 shows the MTT-reducing ability of MDCK cells following the reparative assay (see Methods section for details). In the positive group, most cells remain adhered to after exposure to hydrogen peroxide. However, they have several intracellular vacuoles, which are not seen in cells not exposed to hydrogen peroxide (data not shown). Upon LMG treatment, the MTT was reduced by approximately 20%, like the results from the positive control. Hence, LMG did not show cell damage repairing effect.

#### 3.4.2. Protective Effect Assay Results

**Figure 9:**
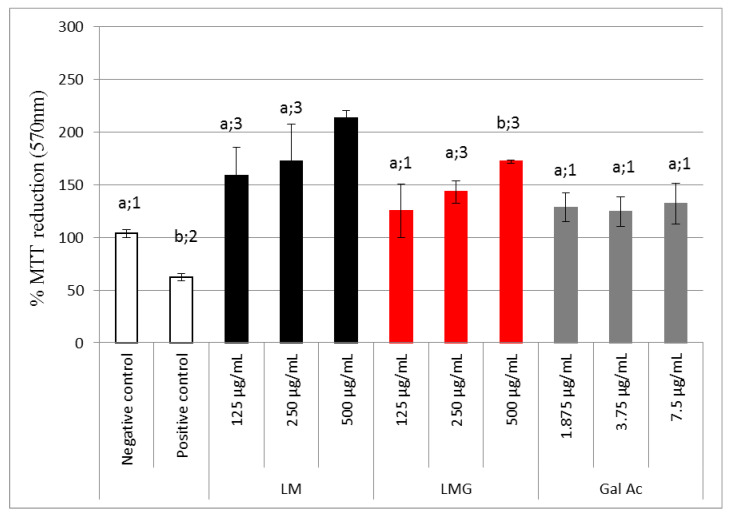
MTT-reducing activity of MDCK cells treated with H_2_O_2_ together with LMG for 24 h (protective effect). Values are expressed as mean ± standard deviation. Different numbers represent a significant difference between controls and LMG (*p* < 0.05). Gal Ac—gallic acid.

The MTT reduction capacity of MDCK cells treated with different LM or LMG and hydrogen peroxide concomitantly is illustrated in Figure 9. Here, all samples showed at least a 100% reduction in MTT (like the negative control). LMG was able to stimulate MDCK cells to reduce MTT, achieving up to a 170% reduction in MTT at a concentration of 500 µg/mL.

Cells incubated with LMG also showed the capacity to promote the reduction of MTT more efficiently than those cells incubated with H_2_O_2_ (positive control) or serum (negative control) (Figure 9). LMG was thus able to prevent damage caused by H_2_O_2_ at all tested concentrations. However, this effect was more pronounced at higher concentrations (e.g., 500 µg/mL LMG).

Phenolic compounds cannot exert their antioxidative effect on cells directly for several reasons [22,72]. First, these compounds are not absorbed well by the human/animal digestive system, and a great portion of ingested phenolics does not even enter the bloodstream.

Second, upon entering the circulatory system, the metabolites are formed from these phenolic compounds, which may have quite different antioxidant properties. Moreover, unconjugated gallic acid was also shown to display a lower reducing power as discussed previously.

One way to improve the antioxidant effect of phenolics may be via increasing their absorption levels. The intake of phenolic compounds in the form of fruits or vegetables (mainly in the form of conjugates with sugars such as glucose) was previously shown to facilitate their absorption [73]. Increased levels of antioxidant activity were observed in the blood plasma upon consumption of oak extract containing gallic acid, elargic acid, gallic acid oligomers, and sugars [74].

Another way of improving the oxidative activity of phenolic compounds is conjugation with polysaccharides. Upon conjugation, antioxidant phenolic compounds are prevented from being metabolized in the liver. Instead, the phenolic compounds enter blood circulation in the form of conjugates and then show biological activity [22]. In addition, conjugated polysaccharides, which may not have any activity in vitro, may show in vivo antioxidative activity, as they are not degraded by metabolic processes in the body.

Seaweed polysaccharides can be used alone or in combination with other natural substances as biomaterials in drug delivery systems [75]. These polysaccharides are biocompatible, biodegradable, obtained from natural sources in a reproducible way, and show low immunogenicity. The conjugation of drugs with polysaccharides also offers several advantages, such as the formation of an amphiphilic conjugate to increase solubility, retention in the gastrointestinal tract for higher absorption, and prevention of being metabolized in the liver to retain biological activity [21,23]. Laminarins constitute a type of polysaccharide obtained from seaweeds and are used as carriers in drug delivery systems for cancer therapy [45,76].

These results indicate that LMG, in addition to its antioxidative effects, may have improved absorption properties as well. Nonetheless, further in vivo tests are necessary to confirm this hypothesis.

#### 3.4.3. Effect of LMG on the Survival and Cell Cycle of MDCK Cells

**Figure 10:**
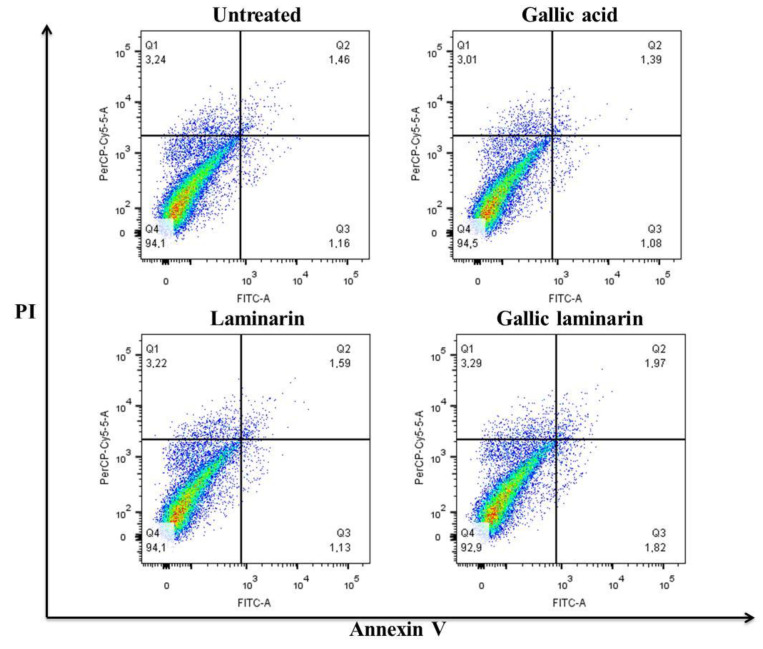
Flow cytometry analysis of MDCK cells stained with annexin (*x*-axis) and propidium iodide(PI) (*y*-axis).

**Figure 11:**
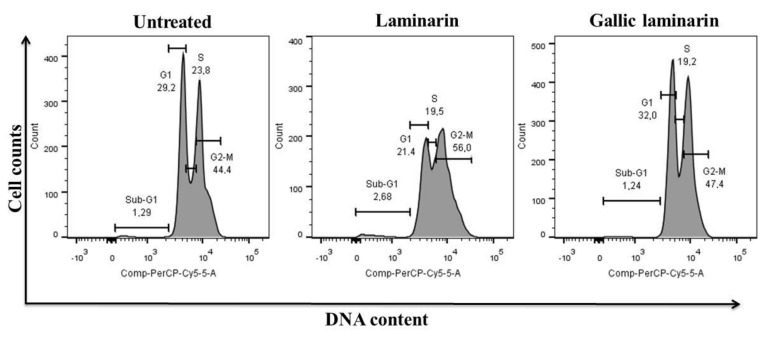
Flow cytometric analysis of MDCK cells stained with PI (*y*-axis) shows the distribution of cells in each cell cycle. Sub-G1 population: indicates cell death from DNA damage; G1 population indicates live cells in G1 phase, population S indicates cells going through the synthesis phase, and G2/M indicates cells in the G2 phase proceeding toward cell division.

Toxicity is an important consideration for possible pharmacological application and/or potential use of a compound in functional food products. Therefore, cell viability tests were performed to determine the toxicity of LMG on MDCK cells as well via cytometry (Figure 10) and cell cycle analyses (Figure 11). Figure 10 shows that 94.1% of untreated cells and 92.4% of cells treated with LMG (0.125 mg/mL) were not stained with annexin and propidium iodide. Hence, LMG was not cytotoxic.

Figure 11 shows that after treatment with LMG (125 µg/mL), 1.24% of cells are in sub-G1 phase, similar to that observed with cells from the control group (1.29%). The percentage of cells treated with LMG in the other cell cycle phases differed from those observed in the control group. However, this difference was not significant. These data indicate that LMG, despite stimulating the ability of cells to reduce MTT, has no proliferative effect on cells.

## 4. Conclusions

In this study, we synthesized three laminarin derivatives, sulfated laminarin (LMS), carboxylated laminarin (LMC), and gallic acid-laminarin (LMG), from a 12.4 kDa laminarin (LM) extracted from *L. variegata* seaweed. The chemical modification of LM promoted slight changes in its MW. The modifications were confirmed by chemical analysis as well. The presence of sulfate groups in LMS was also confirmed by gel electrophoresis. LM and its derivatives showed neither hydroxyl or superoxide radical scavenging nor ferrous chelating activities. Sulfation of LM decreased the copper-chelating activity and the reducing power of LM. LMC performed better as an antioxidant than unmodified LM in the copper chelation test. LMG, on the other hand, showed the highest activity in copper chelation, reducing power, and total antioxidant capacity assays. Gallic acid conjugation was also confirmed by FTIR, ^1^H-NMR, and 2D-NMR spectroscopy analyses, and signals of gallic acid binding to LM molecules were observed. LMG did not induce death or alter the cell cycle of MDCK cells. LMG also protected MDCK cells from oxidative damage caused by hydrogen peroxide. These results suggest that LMG is a potential antioxidant agent that can be used as a drug or functional food ingredient.

## Figures and Tables

**Table 1:** Chemical contents and monosaccharide composition of LM, LMS, LMC, and LMG.

Samples	Proteins (%)	Sulfate (%)	Phenolic Acids (%)	Monosaccharide Composition (%)			
				Glc	Gal	GlcA	Fuc
LM	0.2 ± 0.01	nd	0 ± 0	100	nd	nd	nd
LMS	nd	1.3 ± 0.34	0 ± 0	100	nd	nd	nd
LMC	nd	nd	0 ± 0	88.3	nd	11.7	nd
LMG	0.4 ± 0.04	nd	1.5 ± 0.2	100	nd	nd	nd

LM: Laminarin; LMS: sulfated laminarin; LMC: DBD modified laminarin; LMG: gallic acid-laminarin; nd: not detected; Glc: Glucose; Gal: Galactose; GlcA: Glucuronic acid; and Fuc: fucose.

**Table 2:** Infrared spectra of LM and LMG (positions of the peaks*).

Chemical Group	O–H	C–H	C=O of Ester (Gallic Acid)	C-O-C	C-O	β- Configuration
LM	3402	2924	-	1078	1041	876
GLM	3396	2927	1731	1076	1038	891

*All peaks are presented as cm^−1^.

**Table 3:** Chemical shift assignments of the NMR spectra of LM from *L. variegata*.

Unit	Structural Unit	Chemical Shifts, δ (ppm) ^a^							Ref. ^b^
		H1	H2	H3	H4	H5	H6	H6′	
		C1	C2	C3	C4	C5	C6		
**A**	→3)-β-D-Glcp-(1→	4.81	3.62	3.83	3.57	3.56	3.97	3.78	[66]
		101.5	72.2	83.7	67.2	74.8	59.9		
**B**	→3,6)-β-D-Glcp-(1→	4.55	3.36	3.60	3.46	3.74	4.25	3.92	[67]
		101.8	72.3	83.7	68.8	73.6	67.9		

^a^ Chemical shifts are referred to internal standard trimethylsilyl propionic acid (δ = 0.00 ppm). ^b^ References are used to elucidate structural units based on their similar chemical shifts.
